# Mechanisms and research progress of insect-derived medicines in intervention for ischemic stroke

**DOI:** 10.1016/j.ibneur.2025.11.001

**Published:** 2025-11-04

**Authors:** Chenxi Xu, Yunxiang Guan

**Affiliations:** a Department of Encephalopathy, The First Affiliated Hospital of Henan University of Traditional Chinese Medicine, Zhengzhou, Henan, China; b The First Clinical Medical School, Henan University of Chinese Medicine, Zhengzhou, Henan, China

**Keywords:** Traditional Chinese medicine, Insect-derived medicines, Cerebral infarction, Clinical application

## Abstract

Stroke is a severe neurological disorder caused by the rupture or blockage of blood vessels, leading to significant mortality and disability. Approximately 87 % of cases can be attributed to ischemic stroke. In China, ischemic stroke is the leading cause of death among adults. Current treatments for cerebral infarction mainly include thrombolysis, antiplatelet therapy, anticoagulation, and vasodilation. However, their efficacy in preventing or mitigating primary brain injury remains inadequate. In recent years, traditional Chinese medicine (TCM) has garnered increasing attention due to its proven efficacy and low toxicity. Numerous studies report that TCM formulas, extracts, and compound preparations exhibit pro-angiogenic activity. Insect-derived medicines, known for their actions in detoxification, eliminating pathogens, breaking blood stasis, resolving blood stasis, dispelling wind, and unblocking collaterals, show favorable efficacy in treating ischemic stroke. This paper summarizes several insect-derived medicines and their active components, elucidates their mechanisms of action in antithrombosis, neuroprotection, anti-inflammation, and antioxidation, and discusses current clinical applications and future research directions.

Ischemic stroke, a cerebrovascular event, arises from distinct etiologies including large and small vessel thrombosis, cardioembolism or artery-to-artery embolism, systemic hypoperfusion, and less commonly, cerebral venous thrombosis (Shao et al., 2022). It is a cerebrovascular disease with high disability and mortality rates, often occurring suddenly and unexpectedly (Tu et al., 2023). Thrombosis, the formation of intravascular blood clots, is a major cause of cerebral infarction. The process of clot formation is closely related to the activation of the coagulation cascade, platelet aggregation, and fibrinogen formation (Garmo et al., 2023). Treatment options for thrombosis include anticoagulation, antiplatelet aggregation, and antifibrotic therapy. Treatments for cerebral infarction include intravenous thrombolysis (IVT), endovascular thrombectomy (EVT), and antithrombotic therapy. However, due to time window limitations, IVT and EVT are applicable to only a minority of patients, and their efficacy remains suboptimal, with inevitable risks of hemorrhage and high recurrence rates (Mukherjee and Chattopadhyay, 2022). Antithrombotic therapy employs drugs to prevent thrombus formation or growth, including antiplatelet agents (e.g., aspirin, clopidogrel, ticagrelor) and anticoagulants (e.g., heparin, warfarin, dabigratran) (Greco et al., 2023). With the global population aging, the incidence of stroke is expected to rise, and direct medical costs related to stroke are projected to triple between 2012 and 2030 (Ovbiagele et al., 2013). Given these constraints, the pursuit of innovative treatments has accelerated, bringing the value of herbal formulations to the forefront of drug discovery. Consequently, herbal therapy is now established as a safe and effective option within the investigational realm of ischemic stroke treatment. TCM classifies stroke as "Zhong Feng" ment Zhu et al. (2022a); Ahmadzadeh et al. (2025). After millennia of use and numerous studies, it has been demonstrated that certain traditional Chinese medicines (TCM) and natural drugs offer protection against ischaemic stroke with minimal side effects (Zhu et al., 2021a, Wang et al., 2021, Zhu et al., 2022b). The known mechanisms underlying these anti-ischaemic treatments include inhibiting oxidative stress, apoptosis and inflammatory responses (Zhu et al., 2021b, Liu et al., 2016, Li et al., 2020), as well as promoting angiogenesis (Zhu et al., 2021c, Wang et al., 2020) and neurogenesis (Zhu et al., 2021d, Zhang et al., 2018). These effects can be attributed to the main active compounds present in TCM. Thus, in stroke treatment, insect-derived medicines—known for their dual actions of attacking pathogens and reinforcing healthy qi, resolving blood stasis, dispelling wind, and unblocking collaterals—are frequently used. Recognized as important sources of anticoagulant active substances (Atanasov et al., 2021), insect-derived medicines exert multi-target, multi-link, multi-system, and multi-pathway effects, offering unique advantages in disease treatment. This article provides an overview of the mechanisms and recent research progress of insect-derived medicines in intervening in ischemic stroke.

## 1 Theoretical basis of insect-derived medicines in TCM

### 1.1 Understanding insect-derived medicines

#### 1.1.1 Theoretical basis in TCM

TCM's use of insect-derived medicines dates back 4000 years. *Shennong's Classic of Materia Medica* (*Shen Nong Ben Cao Jing*) systematically documented them, establishing a foundation for TCM theory. Li Shizhen's *Compendium of Materia Medica* (*Ben Cao Gang Mu*) further solidified their role. These medicines retain value and show promise in modern research.

#### 1.1.2 Characteristics and applications

Insect-derived medicines, including dried animals, parts, secretions, and excretions, are a unique TCM component. They offer advantages for complex syndromes and exhibit efficacy against refractory diseases via multi-target interventions. Key pharmacological actions include breaking blood stasis, resolving masses, dispelling wind, and unblocking collaterals, making them effective against wind, phlegm, and stasis-related pathologies. Their blood-activating and stasis-resolving effects align with modern understanding of "insect medicines unblock collaterals." They are readily absorbed. Recent interest stems from their diversity, efficacy, and ease of extraction.

### 1.2 Pathogenesis of Ischemic Stroke from a TCM Perspective

Ischemic stroke refers to brain tissue necrosis caused by ischemia and hypoxia due to cerebral circulatory disorders. It accounts for 87 % of all strokes (Campbell and Khatri, 2020). Its etiology is multifactorial, generally categorized into six aspects: qi, blood, wind, phlegm, deficiency, and fire, acting alone or in combination. The brain is the primary locus, closely related to the heart, liver, spleen, and kidneys. Qi-blood deficiency or liver-kidney yin deficiency is the root cause, while wind, fire, phlegm, and stasis are the manifestations. Precipitating factors like fatigue, agitation, sexual excess, or overeating disrupt yin-yang balance and disturb qi-blood dynamics, leading to stroke. Acute-stage treatment focuses on expelling pathogens, using liver-calming, wind-extinguishing, phlegm-resolving, blood-activating, and collateral-unblocking agents. In the convalescent and recovery phases, treatment addresses both deficiency and excess, supporting healthy qi while eliminating pathogens, employing methods like liver-calming, wind-extinguishing, phlegm-resolving, mass-dissolving, liver-kidney tonifying, and qi-blood replenishing.

## 2 Rationale for using insect-derived medicines in ischemic stroke

### 2.1 Advantages of insect-derived medicines

*The Yellow Emperor's Classic of Internal Medicine* (*Su Wen*) states: "For blood repletion, drainage is appropriate." Blood vessel stasis should be treated by activating blood circulation and resolving stasis. Conventional herbal medicines often show limited efficacy, whereas insect-derived medicines excel at activating blood and unblocking collaterals. They primarily enter the liver meridian and the blood aspect (Yao et al., 2015), breaking stasis, activating blood circulation, and restoring normal qi-blood flow to promote healing. Studies indicate that general blood-activating herbs only dissolve the surface of microthrombi (anti-coagulation), while insect-derived medicines penetrate and dissolve the core (stimulating fibrinolysis).

### 2.2 Clinical expertise

The Treatise on Cold Damage Disorders (*Shang Han Lun*) introduced the concept of "collateral pathways," while the Synopsis of the Golden Chamber (*Jin Gui Yao Lue*) established a series of classic formulas, including the Xia Yu Xue Tang and the Rhubarb and Da Huang Zhe Chong Wan. These insect-based formulations are employed to activate blood circulation, resolve stasis, and unblock collateral pathways in the treatment of collateral diseases (Jiaqian et al., 2025). Collaterals govern qi and blood: qi collaterals transport meridian qi, while blood collaterals carry blood. Pathogens entering collaterals generate pathological products like blood stasis, ultimately leading to tangible lesions like atherosclerosis (Tingting et al., 2022).Ye Tianshi emphasized "collaterals function through patency" and "acridity discharges collaterals," highlighting the unique value of insect-derived medicines in chronic, stubborn diseases (Y et al., 2022). Wang Qingren's Bu Yang Huan Wu Tang is still widely used clinically (Gao and Zhu, 2010). A meta-analysis of 39 studies demonstrated that the combination of Bu Yang Huan Wu Tang and conventional therapy resulted in a significant improvement in NIHSS scores and activities of daily living (ADL) in patients with cerebral infarction, in comparison with conventional therapy administered in isolation (Wang et al., 2022). However, it is important to note that the quality of the evidence in some of these studies may be limited by factors such as small sample sizes and potential publication bias. Consequently, the need for further rigorous, large-scale randomised controlled trials (RCTs) is apparent to confirm these findings. Zhang Xichun adeptly used insect-derived medicines, innovatively proposing the theory " insect-derived medicines communicate with qi and blood."

Modern TCM masters have developed unique strategies for using insect-derived medicines. Master Deng Tietao combined Eupolyphaga (*Tu Bie Chong*) and leech (*Shui Zhi*) to enhance thrombolytic effects; Master Zhu Liangchun used leech and earthworm (*Di Long*) to treat cerebral infarction; Master Yan Dexin advocated scorpion (*Quan Xie*) and centipede (*Wu Gong*) to resolve stasis and unblock collaterals.

The pathogenesis of stroke involves overexertion, qi stagnation and blood stasis, emotional imbalance, and accumulated deficiency impairing vitality, leading to meridian obstruction and disorder. While conventional stroke treatment primarily focuses on restoring blood flow, insect-derived medicines may offer a complementary approach by promoting neuroprotection and enhancing the brain's resilience to ischemic injury. This shift in focus could lead to improved long-term outcomes for stroke patients.

## 3 Pharmacological research progress of selected insect-derived medicines

**Fig. 1:**
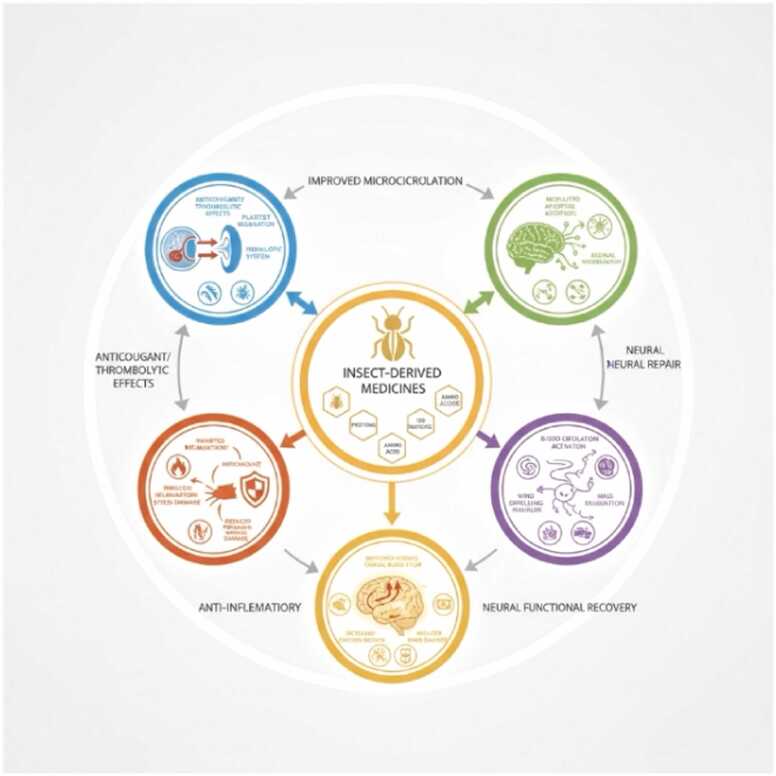
Pharmacological research progress of selected insect-derived medicines.

Advances in modern pharmacology facilitate the identification of active components in insect-derived medicines. Rich in proteins, alkaloids, sterols, polysaccharides, and amino acids, their core mechanisms include: Exerting anticoagulant/thrombolytic effects via specific components; Regulating platelet function and the fibrinolytic system to improve microcirculation; Inhibiting inflammatory cytokine network imbalance and reducing oxidative stress damage; Modulating apoptosis pathways and promoting axonal regeneration and synaptic plasticity. Collectively, they activate blood circulation, resolve stasis, dispel wind, extinguish wind, unblock collaterals, relieve pain, resolve phlegm, and dissipate masses—improving cerebral blood flow, increasing oxygen supply to ischemic areas, reducing brain damage, and promoting neural functional recovery (Zhongmei et al., 2016). Please see Fig. 1.

As most insect-derived medicinal substances possess inherent toxicity and sensitizing potential, with Scorpion and Centipede being toxic, and Leech displaying mild toxicity (Liu et al., 2021), some patients may develop allergic reactions such as pruritus, erythema, or even diarrhea and vomiting during treatment (Feng et al., 2017, Liu et al., 2015, Guo et al., 2019). Strict dosage control is imperative for clinical application, with dosages falling within the safety thresholds stipulated by the Pharmacopoeia of the People's Republic of China. In mild cases, a conservative approach to dosing is recommended, whereas more intensive treatment may be necessary in severe cases. In cases where the standard doses have been proven to be ineffective, particularly in the treatment of critical, acute, or intractable diseases, incremental titration from minimal doses is advised, with the potential for combination with adjuvants to enhance efficacy or mitigate toxicity (Xing et al., 2022) (Table 1).

**Table 1:** Summary of insect-derived medicines.

Drug name	Properties	Meridian	Actions	Pharmacological action
Scorpion (Quan Xie)	Acrid Neutral	Liver	Extinguishes wind, relieves convulsions, unblocks collaterals, relieves pain, attacks toxins, dissipates masses.	Extinguishes wind, relieves convulsions, unblocks collaterals, relieves pain, attacks toxins, dissipates masses.
Earthworm (Di Long)	Salty, Cold;	Liver Spleen Bladder	Clears heat, calms fright, unblocks collaterals, relieves wheezing, promotes urination	Dissolves thrombi, improves cerebrovascular microcirculation, and exerts neuroprotection by modulating JNK1 and PI3K/Akt pathways.
Centipede (Wu Gong)	Warm, acrid	Liver	Extinguishes wind, relieves convulsions, unblocks collaterals, relieves pain, attacks toxins, dissipates masses.	lower plasma cholesterol, triglycerides, and LDL while increasing HDL, improving hemorheology, inhibiting lipid oxidation, and regulating ET−1 and NO levels
Leech (Shui Zhi)	Neutral, salty, bitter	Liver	Breaks blood stasis, unblocks meridians, expels stasis, dissipates masses	naturally regenerate the central nervous system after injury
Silkworm (Jiang Can)	Neutral, salty, acrid;	Liver Lung	Extinguishes wind, relieves convulsions, dispels wind, unblocks collaterals, disperses wind-heat, relieves pain and itching, resolves phlegm, dissipates masses	anticoagulant, antithrombotic, and fibrinolytic effects

### 3.1 Scorpion

Properties**:** Acrid, neutral; enters the liver meridian; toxic. Actions: Extinguishes wind, relieves convulsions, unblocks collaterals, relieves pain, attacks toxins, dissipates masses. According to the Pharmacopeia, the recommended dosage range is between 3 and 6 g (National Pharmacopoeia Commission, 2020). The administration of the substance typically commences with minimal doses, and prolonged use generally demonstrates no toxic reactions. *Yilin Zanyao Tanyuan* (Exploration of Medical Formulas) records scorpion "treats all types of wind" (Fu, 2015). Pre-Tang-Song era, external wind invasion was thought to cause stroke. The Jin-Yuan period emphasized "internal wind". Scorpion treats wind-induced stroke by extinguishing wind and stopping convulsions. Scorpion venom exhibits potent anticoagulant, antithrombotic, and profibrinolytic effects, primarily attributed to its rich content of phospholipases, metalloproteases, and serine proteases (XU, 2010, Hmed et al., 2013, Aloulou et al., 2018, Cordeiro et al., 2018). Phospholipases act as hemolytic agents and contribute to platelet aggregation inhibition, while metallo- and serine proteases modulate cytokine production and complement activation, further influencing the thrombotic process (Aloulou et al., 2018, Cordeiro et al., 2018). While some studies have identified non-protein components like adenosine with anticoagulant properties (Thien et al., 2017), the peptide HsTx2, isolated by Tao Jian et al (Tao et al., 2021)., stands out for its direct anti-stroke effects in rat models. HsTx2 reduced infarct size and improved behavioral deficits by activating the MAPK signaling pathway, suggesting a neuroprotective mechanism beyond simple thrombolysis.

However, the clinical relevance of these findings remains unclear, as the specific concentration of HsTx2 required for efficacy and its potential toxicity in humans have not been fully investigated. Animal experimental doses should be carefully considered for clinical feasibility. Furthermore, while scorpion venom's anti-inflammatory properties are promising, the complex interplay between inflammation and stroke pathology warrants further investigation to determine the optimal timing and dosage for therapeutic intervention. Scorpion is often used in combination with other herbs, such as centipede and earthworm, to enhance its therapeutic effects. However, the compatibility and potential interactions between these herbs need to be further investigated to ensure safety and efficacy. Compared to earthworm extract, which primarily promotes fibrinolysis, scorpion venom's multifaceted action on platelet aggregation, inflammation, and potentially neuroprotection, suggests a broader therapeutic potential, particularly in the early stages of ischemic stroke. However, the risk of allergic reactions and neurotoxicity associated with scorpion venom necessitates careful consideration and further research to identify safer and more targeted delivery.

### 3.2 Earthworm

Properties: Salty, cold; enters the liver, spleen, and bladder meridians. Actions: Clears heat, calms fright, unblocks collaterals, relieves wheezing, promotes urination. First recorded in *Shennong's Classic of Materia Medica*. A plethora of research has indicated that earthworms possess pharmacological properties, including the ability to inhibit platelet aggregation, improve cerebral blood flow, protect neurons against apoptosis and oxidative damage, scavenge free radicals, and reduce inflammation (Wang et al., 2019). Earthworm-derived lumbrokinase is a bioactive component with significant fibrinolytic activity, demonstrated to dissolve fibrin clots effectively (Dong et al., 2019). This thrombolytic action improves cerebrovascular microcirculation and provides neuroprotection, potentially through modulation of the JNK1 and PI3K/Akt pathways. Lumbrokinase exhibits specificity in hydrolyzing both fibrin-rich and fibrin-poor fibrinogen, leading to the direct dissolution of fibrin and subsequent thrombus breakdown (Qiuping et al., 2021). A critical advantage is its fibrin specificity, which minimizes the risk of excessive bleeding. The mechanism involves both direct fibrin dissolution and the conversion of plasminogen to plasmin via endogenous t-PA induction, further contributing to a reduced hemorrhage risk (Yang et al., 2021). Thrombolytic assays show earthworm protein hydrolysates (HEPLPEP, EYPLPEP) inhibit thrombosis and degrade thrombi, prolonging thrombin clotting time (Feng et al., 2022). Earthworm extract also acts on PI3K/Akt, regulating TNF-α, ERK1/2, and Akt to inhibit platelet aggregation/activation and improve microcirculation. In vitro thrombolytic assays using earthworm protein hydrolysates (HEPLPEP, EYPLPEP) have shown thrombosis inhibition, thrombus degradation, and prolonged thrombin clotting times (Yang et al., 2021). Furthermore, earthworm extract influences the PI3K/Akt pathway, regulating TNF-α, ERK1/2, and Akt, ultimately inhibiting platelet aggregation and activation and improving microcirculation. However, a direct comparison of their in vivo efficacy and safety profiles, particularly in models of acute thrombosis, is lacking. This represents a crucial area for future research. Specifically, studies should investigate the optimal timing and combination of these agents – could scorpion venom be used prophylactically to prevent clot formation, followed by lumbrokinase to dissolve any clots that do form? Furthermore, the specific subtypes of phospholipases in scorpion venom responsible for the antithrombotic effect need to be identified and characterized, and the potential for synergistic effects between these phospholipases and lumbrokinase needs to be explored. Finally, research should focus on developing targeted delivery systems for lumbrokinase to enhance its thrombus specificity and minimize systemic exposure, thereby further reducing the risk of bleeding complications. Understanding the nuances of their mechanisms and conducting comparative studies will be essential for determining their respective clinical roles and potential for combined therapeutic strategies. Further research is needed to elucidate the long-term effects and potential immunogenicity of both compounds.

Recent research shows (Zhao et al., 2025) Earthworm extract (EWE) significantly modulated the inflammatory response in lipopolysaccharide (LPS)-induced microglia by suppressing pro-inflammatory cytokines and stimulating anti-inflammatory cytokines. Furthermore, EWE-conditioned medium (EWE-CM) enhanced the viability and tube-forming capacity of HCMEC/D3 cells, suggesting promotion of angiogenesis via the Ang1/Tie2/Ang2 pathway. In vivo, EWE administration in MCAO/R-induced mice significantly ameliorated neurological deficits, reduced infarct volumes, and suppressed microglial cell activation. These findings suggest that EWE modulates microglial cell M1/M2 polarization and enhances angiogenesis, potentially through the Ang1/Tie2/Ang2 pathway, following cerebral ischemia. These effects underscore the potential of EWE as a therapeutic strategy for stroke.

### 3.3 Centipede

Properties: Warm, acrid; toxic; enters the liver meridian. Actions: Extinguishes wind, relieves convulsions, unblocks collaterals, relieves pain, attacks toxins, dissipates masses. The Pharmacopoeia stipulates a dosage of 3–5 g (National Pharmacopoeia Commission, 2020).First recorded in *Shennong's Classic of Materia Medica* (Jianbing, 2018*)*. Zhang Xichun noted its potent "penetrating and dispersing power… opening all areas of qi-blood stagnation." Centipede venom peptides/proteins demonstrate promising antithrombotic effects, offering a complex mechanism beyond simple anticoagulation. The venom contains a variety of acidic proteins that influence lipid metabolism, reducing plasma cholesterol, triglycerides, and LDL while increasing HDL, thereby improving hemorheology, inhibiting lipid oxidation, and regulating endothelin-1 (ET-1) and nitric oxide (NO) levels (Wu et al., 2009). NO, synthesized from L-arginine by eNOS, plays a crucial role in preventing thrombosis by inhibiting platelet aggregation and smooth muscle proliferation (Freedman and Loscalzo, 2003). The FeCl₃-induced carotid thrombosis mouse model is frequently used to assess antiplatelet efficacy. In a study by Wonhwa et al. (2016), control mice exhibited rapid thrombus formation following FeCl₃-induced endothelial injury (8.5 ± 0.9 min), whereas centipede extract-treated mice showed delayed thrombus growth and reduced overall thrombosis at 60 min. Standard in vitro assays, including prothrombin time (PT), activated partial thromboplastin time (APTT), fibrin polymerization assays, and platelet aggregation assays, are used for antithrombotic evaluation. Centipede extract prolonged PT/APTT, significantly reduced the rate of fibrin polymerization, and inhibited platelet aggregation, without exhibiting cytotoxicity to human umbilical vein endothelial cells (HUVECs). In vitro and ex vivo studies have further demonstrated that centipede-derived fibrinolytic enzymes inhibit Factor Xa (FXa) and thrombin generation in HUVECs, thereby suppressing coagulation pathways and TNF-α-induced plasminogen activator inhibitor-1 (PAI-1) secretion and subsequent platelet aggregation. Therefore, centipede extracts and small peptides possess significant anticoagulant and antiplatelet effects, suggesting potential therapeutic applications for conditions such as cerebral infarction.

However, current research lacks a comprehensive understanding of the specific peptides or proteins responsible for each observed effect. While the study by Wonhwa et al. provides valuable in vivo data, the specific venom components responsible for the observed thrombus reduction remain unidentified. Further fractionation and characterization of centipede venom are needed to isolate and identify the key antithrombotic peptides/proteins. Furthermore, the relative contribution of lipid modulation, NO regulation, and direct effects on coagulation factors to the overall antithrombotic effect needs to be elucidated. Are the lipid-lowering effects a prerequisite for the antithrombotic activity, or do they act independently? Crucially, the long-term safety and efficacy of centipede venom extracts or purified peptides require thorough investigation in preclinical models, including assessment of potential immunogenicity and off-target effects. Moreover, a direct comparison of the antithrombotic efficacy of centipede venom with established antithrombotic drugs, such as aspirin or clopidogrel, is essential to determine its potential clinical value. Future studies should also explore the potential for synergistic effects between centipede venom components and existing antithrombotic therapies. Finally, given the complexity of centipede venom, research should investigate the potential for developing synthetic peptides mimicking the active sites of the venom's antithrombotic components. This could lead to more targeted and safer therapeutic agents.

### 3.4 Leech

Properties: Neutral, salty, bitter; slightly toxic; enters the liver meridian. Actions: Breaks blood stasis, unblocks meridians, expels stasis, dissipates masses. Characteristics: Potent and targeted, effectively dispels wind, unblocks collaterals, activates blood (Pu, 2015). The Pharmacopoeia stipulates a dosage of 1–3 g (National Pharmacopoeia Commission, 2020). First recorded in *Shennong's Classic of Materia Medica*. Modern studies (Li et al., 2018) have identified two primary classes of bioactive components in leeches: 1) Macromolecules, such as hirudin-like proteins/peptides; and 2) Small molecules, including pteridines. Leeches have a long and well-documented history in anticoagulation. Thrombin (THR) plays a central role in the coagulation cascade, converting fibrinogen to fibrin, activating FXIII→FXIIIa, and activating cofactors V/VIII/XI to amplify its own generation (Davie et al., 1991, Richardson et al., 2000). Therefore, THR inhibition is a critical strategy for disrupting thrombosis (Gailani and Broze, 1991). Huang Qiuyang et al (Huang et al., 2020). were the first to isolate a novel THR-inhibitory peptide (SYELPDGQVITIGNER) from leech. Molecular docking studies revealed strong binding affinity to THR's active site, suggesting significant therapeutic potential. Leech extract has been shown to inhibit THR-induced tissue factor (TF) expression in vascular endothelial cells (VECs) and counteract THR's suppression of tissue factor pathway inhibitor (TFPI) release (Guofeng et al., 2017),linking these effects to its observed anticoagulant and antithrombotic properties and underscoring its potential significance in the prevention of cardiovascular and cerebrovascular diseases. However, the clinical application of leech-derived products is limited by the risk of bleeding complications, allergic reactions, and potential transmission of blood-borne pathogens. In addition to THR inhibitors, leeches also contain elastase inhibitors, thrombin inhibitors, and other enzymes/proteins, all contributing to its blood-activating and stasis-resolving actions. This multifaceted mechanism of action provides a multi-component, multi-target, multi-pathway advantage in the prevention and treatment of ischemic stroke. Leech-derived hirudin directly inhibits thrombin, offering a seemingly more targeted approach compared to agents like silkworm extract, which modulates the broader coagulation cascade. However, this specificity presents both an advantage and a potential limitation.

While hirudin's direct THR inhibition is effective in situations where thrombin is the primary driver of thrombosis, its singular focus might reduce its efficacy in more complex thrombotic environments where other coagulation factors or inflammatory mediators contribute significantly to clot formation. A critical area for future research is to fully characterize the synergistic interactions between the various bioactive components of leech extract. For example, how do the pteridines contribute to the overall antithrombotic effect, and do they interact with hirudin or other enzymes to enhance its activity? Furthermore, the long-term effects of leech-derived compounds on endothelial function and vascular remodeling require further investigation. Specifically, do these compounds promote endothelial repair or prevent the development of chronic vascular inflammation? While hirudin has been extensively studied, more research is needed to identify and characterize novel hirudin-like peptides with potentially improved pharmacokinetic properties or reduced immunogenicity. A direct comparison of the efficacy and safety of leech-derived products with existing anticoagulant therapies, such as heparin or direct oral anticoagulants (DOACs), is also essential to determine their potential clinical niche. Finally, developing novel drug delivery systems to target leech-derived antithrombotic agents specifically to the site of thrombus formation could improve their efficacy and minimize the risk of bleeding complications. Understanding the nuances of the leech's complex mechanism of action and conducting comparative studies will be crucial for determining their clinical roles and potential for novel therapeutic strategies. Further research should be directed towards the isolation, characterization, and optimization of these compounds for targeted drug delivery to maximize efficacy and minimize off-target effects.

### 3.5 Silkworm

Properties: Neutral, salty, acrid; enters the liver and lung meridians. Actions: Extinguishes wind, relieves convulsions, dispels wind, unblocks collaterals, disperses wind-heat, relieves pain and itching, resolves phlegm, dissipates masses. First recorded in *Shennong's Classic of Materia Medica*. *Yu Qiu Yao Jie* (Jade Axe Medicinal Explanations) notes its ability to "activate collaterals, unblock meridians, dispel wind, open impediments (Mo et al., 2016)." Acridity moves qi; saltiness enters blood; it unblocks collaterals, dispels wind-phlegm, and generates new blood to move qi. Modern pharmacological studies have confirmed the anticoagulant, antithrombotic, and fibrinolytic effects of silkworm extracts (Liu et al., 2025). These extracts prolong activated partial thromboplastin time (APTT), prothrombin time (PT), and thrombin time (TT), primarily by inhibiting Factor XII (FXII) activity. While antithrombin-III (AT-III) is a crucial endogenous anticoagulant, research indicates that the anticoagulant substances in silkworm function independently of AT-III levels, maintaining efficacy even in thrombotic or AT-III-deficient conditions (Zhang et al., 2020). This suggests a potentially superior clinical value in the treatment of AT-III-deficient diseases compared to AT-III dependent anticoagulants like heparin. Wang et al. (2024) investigated the pharmacodynamics of silkworm extract in rat ischemia-reperfusion models, demonstrating that higher extract concentrations reduced neuronal damage and promoted neovascularization. This resulted in improved blood supply to ischemic areas and mitigation of post-ischemic injury, supporting the potential application of silkworm extracts in promoting recovery from cerebral infarction.

However, the precise mechanism by which silkworm extracts exert their FXII inhibitory effect remains unclear. Further research is needed to isolate and identify the specific compounds responsible for this activity and to determine their binding affinity and selectivity for FXII. Furthermore, while the AT-III independence is a potentially significant advantage, more comprehensive studies are required to fully elucidate the downstream effects of silkworm extract on the coagulation cascade in both AT-III-replete and AT-III-deficient states. Specifically, how does the extract impact the activation of other coagulation factors, such as Factor XI, and what is its effect on thrombin generation? The study by Wang Shanshan et al. provides promising evidence for neuroprotective effects, but further investigation is needed to determine the optimal dosage and timing of silkworm extract administration in the context of ischemia-reperfusion injury. In addition, the long-term safety and efficacy of silkworm extract in preventing recurrent stroke needs to be evaluated in preclinical models. A direct comparison of silkworm extract with existing stroke therapies, such as thrombolytic agents (e.g., tPA) and antiplatelet drugs (e.g., aspirin), is essential to determine its relative efficacy and potential for synergistic effects. Finally, exploring the potential for targeted delivery of silkworm-derived compounds to the ischemic brain could enhance their therapeutic efficacy and minimize systemic side effects. Understanding the precise mechanisms of action, optimizing dosing regimens, and conducting comparative studies will be crucial for determining the clinical potential of silkworm extracts in the treatment of stroke.

### 3.6 Broader insights from animal-derived peptides

While the primary focus of this thesis is on insect-derived medicines, insights from related animal sources, such as amphibian skin secretions, offer valuable comparative perspectives on the neuroprotective mechanisms in ischemic stroke. Amphibians, like insects, produce bioactive peptides with potent antioxidant and anti-apoptotic properties, which have often evolved for defense against environmental stressors (Li et al., 2023). A recent study exemplified this with OL-FS13, a novel 13-amino-acid peptide isolated from the odorous frog *Odorrana livida* via cDNA library screening (Liu et al., 2022). In rat models of cerebral ischemia-reperfusion (I/R) injury and PC12 cell oxygen-glucose deprivation/reoxygenation (OGD/R) models, OL-FS13 (administered post-I/R at 10 μg/kg i.p.) significantly reduced infarct volume (by approximately 50 % via TTC staining), improved neurological scores (mNSS), and ameliorated histological damage (H&E and Nissl staining), with no observed toxicity at effective doses.

Mechanistically, OL-FS13 enhances the nuclear translocation of Nrf2, upregulating HO-1 expression and boosting antioxidant enzymes (SOD and CAT levels increased by 1.5–2-fold, while MDA and LDH decreased), thereby countering oxidative stress, a pathway shared with insect-derived peptides such as scorpion venom chlorotoxin, which also modulates Nrf2 to mitigate ROS in stroke (Zhu et al., 2022b). Additionally, OL-FS13 inhibits ER stress by downregulating GRP78, phosphorylating IRE1α, and suppressing the TRAF2/JNK axis, restoring the Bcl-2/Bax balance, and reducing cleaved caspase-3 (apoptosis markers reduced by 40–60 %) (Liu et al., 2022). The use of the Nrf2 inhibitor ML385 abolished these effects, confirming Nrf2 as the central mediator. A follow-up study further elucidated OL-FS13's regulation of miRNA networks, revealing the downregulation of miR-21–3p (via multi-omics sequencing) and subsequent upregulation of its target CAMKK2, activating the AMPK/Nrf2 axis to restore ATP homeostasis and inhibit apoptosis in OGD/R PC12 cells and CI/R rats. Overexpression of miR-21–3p (via mimic/agomiR) antagonized these benefits, increasing infarct volume and neurological deficits, whereas CAMKK2 inhibition (STO-609) blocked Nrf2 activation, confirming the miR-21–3p/CAMKK2/AMPK/Nrf2 cascade as pivotal (Liu et al., 2023). This miRNA-mediated energy regulation parallels insect peptides, such as earthworm lumbrokinase's PI3K/Akt modulation for neuroprotection (Yang et al., 2021) and leech hirudin's anti-apoptotic effects via calcium homeostasis (Guofeng et al., 2017), suggesting conserved mechanisms across phyla for countering ischemia-induced calcium overload and oxidative stress.

Compared to longer amphibian peptides such as OM-LV20 (Yin et al., 2021), OL-FS13's shorter sequence facilitates cost-effective synthesis and post-injury efficacy, mirroring the translational advantages of compact insect peptides (e.g., melittin from bee venom) (Zhongmei et al., 2016). These findings underscore the potential for cross-phyla peptide engineering, where insect motifs could be fused with amphibian stability enhancers to target the oxidative/ER stress and miRNA dysregulation cascades induced by stroke (Liu et al., 2022, Liu et al., 2023).

These findings expand the therapeutic potential of animal-derived peptides beyond insects, offering low-toxicity candidates for adjunctive stroke therapy and underscoring the need for comparative studies on their synergistic use with TCM insect medicines.

## 4 Clinical applications of insect-derived medicines

### 4.1 Proprietary chinese medicines

In recent years, proprietary Chinese medicines (PCMs) primarily composed of insect-derived drugs have been increasingly utilized in the treatment of ischemic stroke. According to the *Clinical Medication Guidelines of the Pharmacopoeia of the People's Republic of China: Chinese Patent Medicines Volume (2020 edition)*, numerous PCMs containing insect-derived components are indicated for stroke management. Examples include: Scorpion-containing: Naoxuekang Capsule, Tongxinluo Capsule, Xiaoshuan Tongluo Capsule. Earthworm-containing: Compound Dilong Capsule, Xingnao Zaizao Pill, Renshen Zaizao Pill. Centipede-containing: Naoshuantong Capsule, Zhongfeng Huichun Tablet. Leech-containing: Maixuekang Capsule, Xueshuan Xinmaining Tablet. Silkworm-containing: Huatan Tongluo Capsule. The use of the above medications is all based on clinical observations.

Despite the extensive utilisation of these PCMs in clinical practice, the substantiation of their efficacy is frequently constrained to observational studies and case series. A paucity of rigorous randomised controlled trials (RCTs) has been conducted to evaluate the effectiveness of these interventions rigorously, and the quality of existing studies is often compromised by methodological limitations. Additionally, the intricate composition of these PCMs poses a significant challenge in identifying the components responsible for their therapeutic effects and in optimising their formulation.

### 4.2 Clinical experience of TCM masters

TCM masters employ insect-derived medicines distinctively: Master Zhu Liangchun: Advocated insect-derived medicines (e.g., leech, earthworm in self-formulated "Nao Luo Fu Yuan Tang") for "stasis-toxin obstructing collaterals" in chronic disease, emphasizing combined pathogen-attacking and qi-tonifying strategies. Master Yan Dexin: Viewed ischemic stroke as "stasis obstructing the clear orifices," requiring blood-breaking and collateral-unblocking to restore consciousness. Modified "Tong Qiao Huo Xue Tang" with leech and silkworm. Master Deng Tietao: Attributed ischemic stroke to "internal liver wind and phlegm-stasis obstructing collaterals," prioritizing insect-derived medicines for liver-calming and wind-extinguishing, supplemented by spleen-fortifying and phlegm-resolving. Used modified "Bu Yang Huan Wu Tang" with earthworm and leech for qi-deficiency blood-stasis type, highlighting large-dose Astragalus (120 g) to "move qi and thus move blood." The clinical experience of Traditional Chinese Medicine (TCM) masters provides valuable insights into the potential applications of insect-derived medicines. However, it is important to acknowledge that the evidence for these applications is primarily anecdotal and lacks the rigor of controlled clinical trials. The individualisation of treatment based on TCM syndromes introduces a further layer of complexity to the evaluation of efficacy, as it is challenging to standardise treatment protocols and compare outcomes across different patients. The absence of standardised diagnostic criteria and outcome measures serves to further complicate the assessment of treatment effectiveness.

In summary, insect-derived medicines hold irreplaceable clinical value in the TCM diagnostic-treatment system for ischemic stroke. In the future, we hope to have a precise treatment plan for stroke based on traditional Chinese medicine theory, specifically tailored to the individual's TCM syndromes and specific pathological and physiological characteristics, selecting and combining insect medicinal materials. This plan aims to maximize the therapeutic benefits of insect medicines while minimizing the risk of adverse reactions.

## 5 Conclusion

Insect-derived medicines demonstrate unique clinical value in ischemic stroke treatment. Their mechanisms align with core TCM theories—activating blood circulation, resolving stasis, unblocking collaterals, dispelling phlegm, calming the liver, and extinguishing wind—while modern pharmacology confirms multi-target interventions (e.g., antithrombosis, platelet inhibition, coagulation-fibrinolysis regulation). This provides crucial theoretical and practical guidance for TCM-based cerebral infarction management. However, challenges remain: 1) Unclear component-effect relationships: Most insect-derived medicines are used in compounds, but synergistic/antagonistic interactions of components and compatibility rules lack systematic study, hindering precise clinical protocols. 2) Limited clinical positioning: While insect-derived medicines excel in the recovery phase, their application in the acute phase is less developed. However, the discovery of natural peptides like OL-FS13, which show efficacy when administered post-injury, suggests that targeted neuroprotective agents from natural sources could fill this therapeutic gap (Liu et al., 2022)**.** 3) Inadequate safety assessment: While overall clinical efficacy exceeds 85 % (e.g., 91.3 % for Da Huang Zhe Chong Wan), it should be noted that this figure is based on limited and potentially biased data. The potent nature of these medicines may cause gastrointestinal reactions, bleeding tendencies, or allergic reactions in vulnerable patients (e.g., those with a weak constitution or coagulation disorders). A systematic analysis of the incidence of adverse effects is lacking, necessitating the development of individualised risk-warning models. 4) Delayed translation of basic research: Only 6 insect-derived active components (e.g., hirudin, lumbrokinase) have well-defined molecular mechanisms. Pharmacokinetics and metabolites of most compounds remain unstudied. Future studies should focus on identifying the specific bioactive compounds responsible for the therapeutic effects of each insect medicine, elucidating their mechanisms of action at the molecular level, and developing standardized extracts with consistent potency and safety profiles.

Thus, discovering novel bioactive compounds with diverse mechanisms to enhance efficacy and reduce toxicity is essential. Promisingly, advanced technologies (e.g., single-cell sequencing, organoid models) will facilitate breakthroughs in deeper mechanisms like blood-brain barrier regulation and neurovascular unit repair. A closed-loop approach—mechanistic elucidation → clinical validation → safety reassessment—may transform insect-derived medicines from empirical use to precision medicine, offering innovative solutions integrating TCM characteristics with modern scientific connotation for ischemic stroke treatment. In addition, insect-derived drugs can serve as an adjunct to conventional thrombolytic therapy to improve recanalization rates and reduce ischemic damage. They can also be used in post-stroke rehabilitation to promote neural plasticity and functional recovery.

## Patents

None

## CRediT authorship contribution statement

**Xu Chenxi XU:** Writing – original draft, Methodology, Investigation, Formal analysis, Data curation, Conceptualization. **GUAN YunXiang:** Writing – review & editing, Validation, Supervision, Resources, Funding acquisition.

## Funding

This research was funded by Health Commission of Henan Province, grant number 2022ZYBJ06.

## Declaration of Competing Interest

The authors declare the following financial interests/personal relationships which may be considered as potential competing interests: YunXiang GUAN reports financial support was provided by Health Commission of Henan Province. If there are other authors, they declare that they have no known competing financial interests or personal relationships that could have appeared to influence the work reported in this paper.
